# Selective biliary occlusion in rodents: description of a new technique

**DOI:** 10.1515/iss-2021-0044

**Published:** 2022-06-23

**Authors:** Beate Richter, Constanze Sänger, Franziska Mussbach, Hubert Scheuerlein, Utz Settmacher, Uta Dahmen

**Affiliations:** Department of General, Visceral and Vascular Surgery, Experimental Surgery Unit, University Hospital Jena, Jena, Germany; Clinic for General, Visceral and Paediatric Surgery, St. Vincenz Hospital Paderborn, Teaching Hospital of the University of Göttingen, Paderborn, Germany

**Keywords:** cholestasis research, experimental surgery, hepatobiliary remodelling, selective biliary transection

## Abstract

**Background:**

Modern therapy concepts are of limited success in patients with cholestasis (e.g., biliary occluding malignancies). Therefore, we established a new animal model enabling simultaneous investigation of liver regeneration and hepato-biliary remodelling in biliary obstructed and biliary non-obstructed liver lobes.

**Methods:**

Biliary occlusion of different extent was induced in 50 male rats: Ligation and transection of the common bile duct (100% of liver, tBDT, n=25); or of the left bile duct (70% of liver, sBDT, n=25). At postoperative days 1, 3, 7, 14 and 28 we assessed the hepatic histomorphological alterations, proliferative repair, progress of liver fibrosis (HE, BrdU, EvG) and signs of liver regeneration (liver lobe weight gain). In addition, we determined systemic markers of hepatocellular injury (ASAT, ALAT), cholestasis (Bilirubin) and synthetic liver function (INR). The animals were monitored daily (body weight gain, stress score, survival).

**Results:**

All animals survived until the planned date of sacrifice. sBDT induced in the biliary occluded liver lobes similar histomorphological alterations, proliferative repair and progress of liver fibrosis like tBDT. In the biliary non-ligated liver lobes in sBDT animals we noticed a temporarily enhanced biliary proliferation and a persistent low grade liver fibrosis in the periportal area.

**Conclusions:**

Our model of sBDT represents a safe and valid method to induce selective cholestasis. The model enables further comparative investigation of liver regeneration in different extents of occlusive cholestasis (e.g., mimicking biliary occluding malignancies).

## Introduction

The implementation of new multi-staged therapy concepts for advanced liver tumours led to an impressive improvement for patients with primary non-resectable liver malignancies. Still, such modern therapy concepts are of limited success in patients with cholestatic altered liver parenchyma. Even livers with lobar cholestasis without systemic signs of cholestasis repeatedly show insufficient signs of liver regeneration (e.g., volume gain of the future liver remnant, FLR). Thus, the extent of the pre-existing, even if locally limited, cholestasis seems to co-determine the success of the modern therapy concepts [1], [2], [3], [4].

Therefore, we established an experimental model with selective biliary occlusion in order to simulate a locally advanced hilar/intrahepatic biliary occlusion without systemically detectable signs of cholestasis (e.g., biliary occluding malignancies, Klatskin IIb-III°).

We intended to answer specific questions with our study:

– Are the histomorphological alteration of the biliary ligated liver lobes comparable to the already described cholestatic alteration after complete biliary occlusion?
– Is the complex technique of sBDT associated with more complications and less well tolerated than total biliary occlusion?

## Materials and methods

### Experimental design

We performed one experiment with two experimental groups in male Lewis rats (n=50).

This experiment was designed to study the impact of a selective biliary occlusion (sBDT) of 70% of liver volume on liver regeneration and hepatobiliary remodelling in the biliary ligated and the non-ligated liver lobes. We included a group with total biliary occlusion (tBDT, 100%) for comparison.

At five time points (postoperative days (POD): 1, 3, 7, 14, 28) after tBDT or sBDT, the animals (n=5/group) were randomly assigned for sacrifice, and samples of blood and liver lobes were collected for further analyses.

### Animals

All surgical procedures were performed in inbred male rats (Lewis, aged 9–10 weeks, body weight 250–280 g). We obtained the animals from a commercial breeding laboratory (Charles River, Sulzfeld, Germany). We performed the experimental procedures and housing of the animals according to the German Animal Welfare Legislation. The experimental work and housing were approved by the local authorities (Landesamt für Verbraucherschutz Thüringen).

### Surgical techniques

#### Total biliary occlusion (tBDT)

**Figure 1:**
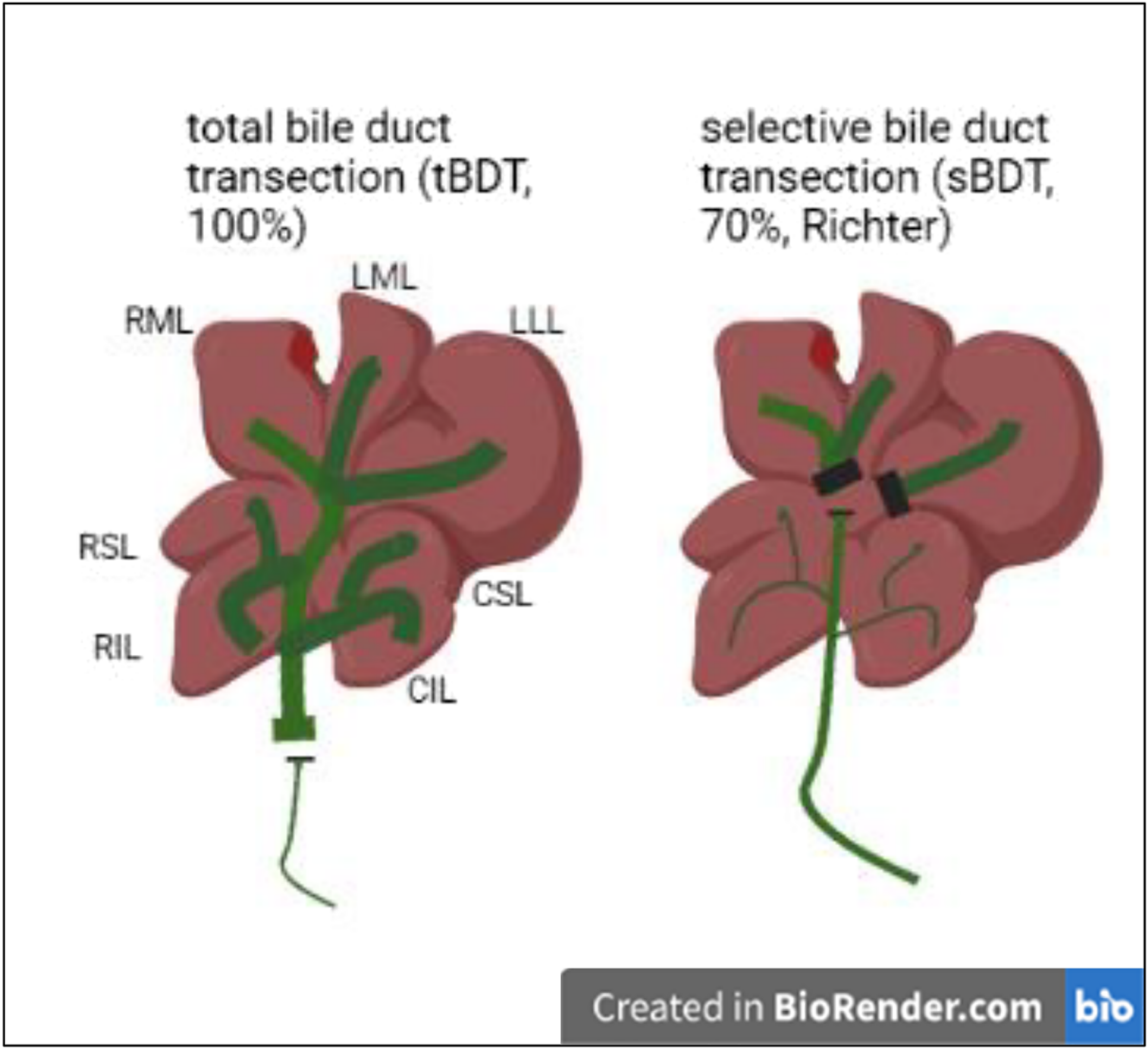
Comparison of the techniques for tBDT and sBDT. tBDT is performed by placing ligatures around the distal part of the main extrahepatic bile duct and transection of the ligated duct segment between the two distal ligatures. tBDT demands medium level experiences in microsurgery. Whereas for sBDT very precise experiences in hepatobiliary microsurgery is needed, since multiple ligatures need to be placed around the bile ducts draining the superior liver lobes (RML, LML and LLL) and several transections of ligated bile duct segments are performed. An operation microscope is mandatory for sBDT. The picture was created using BioRender.com.

We induced total biliary occlusion by ligation and transection of the ligated main extrahepatic bile duct (tBDT) between the middle and distal of three ligatures in 25 animals ca. 1 cm above the pancreas (see Figure 1).

#### Selective biliary occlusion (sBDT)

To induce a selective biliary occlusion in 70% of the liver volume, we placed each two ligatures around the branches of the hilar bile ducts draining the median lobe and the left lateral lobe and around the more distal segment of the confluence of both draining bile ducts (∼ superior part of the main bile duct (see Figure 1)). The median lobe and the left lateral lobe (ML+LLL) represent 70% of the whole liver volume [5, 6]. We transected the ligated bile duct branches between the middle and distal ligatures to prevent biliary leakage and recanalization.

Detailed description of the surgical procedure and postoperative observation and analgetic treatment of the animals are given in supplement.

### Determination of the operation time, body weight gain, liver weights

The operation time was noticed as ”cut-to-stitching-time”, including the time from opening the abdominal cavity until the last stitch of the last suture was finished in minutes (min).

The animals were weighed daily until the end of the observation period. The body weight gain was calculated by dividing the weight of the animal of the dedicated day [g] by the starting body weight [g] of the animal. The liver was weighed after explantation using an analytical balance (BLC-3000, Boeco Germany). Liver body weight ratio was calculated by dividing the weight of the liver [g] by the starting body weight [g] of the animal, respectively.

We included the liver weight data of an untreated male lewis rat (250 g bw, at POD 0: whole liver=10 g, ML+LLL=7 g; RL+CL=3 g) of our laboratory for better understanding of the differences in weight gain of the biliary ligated and non-ligated liver lobes in either group.

### Laboratory measurements

#### Clinical chemistry (liver enzymes and systemic parameters)

Serum was stored at −20 °C until measurement of the transaminases (ALT, AST), parameters for hepatic metabolism (INR, Gluc, Bili, Albumin) and renal function (GFR) using an automated chemical analyser (Bayer Advia 1650, Leverkusen, Germany).

### Histology and immunohistochemistry

We obtained samples from the middle part of every liver lobe assuring evaluation of comparable areas of the liver lobes in all animals. Sections, 4um thick, were cut after paraffin embedding.

Haematoxylin–eosin staining (HE) was used for histologic and morphological analysis of the liver tissue. Elastica van Gieson (EvG) staining was employed for quantification of relative content (relative area per slide) of collagen (Collagen Index) and for assessment of the distribution of fibrosis in relation to anatomical landmarks (Fibrosis Score). Bromodesoxyuridin (BrdU) staining was the basis for detection of the proliferation indices of hepatocytes and cholangiocytes in one sample.

Detailed descriptions of staining methods are listed in supplement. After staining all slides were digitalized using a slide scanner (Nanozoomer, Japan).

#### Quantification of proliferation (BrdU)

The proliferative activity of hepatocytes (BrdU) and the quantification of accumulated fibrous tissue (Collagen-Index, EVG) were determined using the HistoKAt software developed at Fraunhofer MEVIS (Dr. Homeyer, Fraunhofer MEVIS, Bremen, Germany). This software can be trained to recognize certain structures (e.g., cell nuclei) or defined patterns and is suitable for batch analysis. The software was kindly provided by Fraunhofer-Institute (Fraunhofer MEVIS, Bremen, Germany) [7].

Proliferative activity of cholangiocytes was determined by counting BrdU-positive cholangiocytes per bile duct in 10 high power fields (HPF; 40× magn.) of periportal fields and in 10 HPF of intralobular area (“extra-portal ductular reaction”) per slide (using NPG-Viewer).

Detailed descriptions of staining methods are listed in supplement.

#### Quantification of relative content of collagen and elastic fibres (Collagen-Index) and semi-quantitative assessment of the severity of fibrosis (Fibrosis Score) using EVG staining

The Collagen Index was calculated irrespective of the location of the positively stained areas (periportal, pericentral). To assess the severity of fibrosis, we additionally used the established fibrosis staging score according to Blunt modified for rodents by Lo and Gibson-Corley [8–10]. This score reflects location and extent of fibrosis and includes periportal, pericentral and bridging fibrosis and cirrhosis (see Table 1). We assessed 10 HPF (40× magn., EvG staining) of periportal and pericentral areas per slide and animal using the NPD-Viewer. The median of the fibrosis score is given to avoid under- or over-scoring according to Lo and Gibson-Corley [11, 12].

**Table 1:** Modified fibrosis score according to Blunt, Lo and Gibson-Corley [11–13].

Score	Explanation
0	No fibrosis (∼ no positive staining)
1	Periportal fibrosis
2	1 + with pericentral fibrosis
3	2 + with bridging fibrosis
4	Cirrhosis

### Statistical analysis

The data are expressed as mean ± standard deviation (SD) if not indicated otherwise. The data were analysed using SPSS (IBM SPSS 22 for Windows). Type of distribution was determined using the Kolmogorow–Smirnow test (including the correction of significance according to Lilliefors). As the test revealed a non-normal distribution, the data were analysed using non-parametric tests (Kruskal–Wallis Test, Mann–Whitney-U-Test). Differences were considered significant if p-value of less than 0.05 (2-tailed) were obtained.

## Results

### Survival, operation time and recovery of the animals

**Figure 2:**
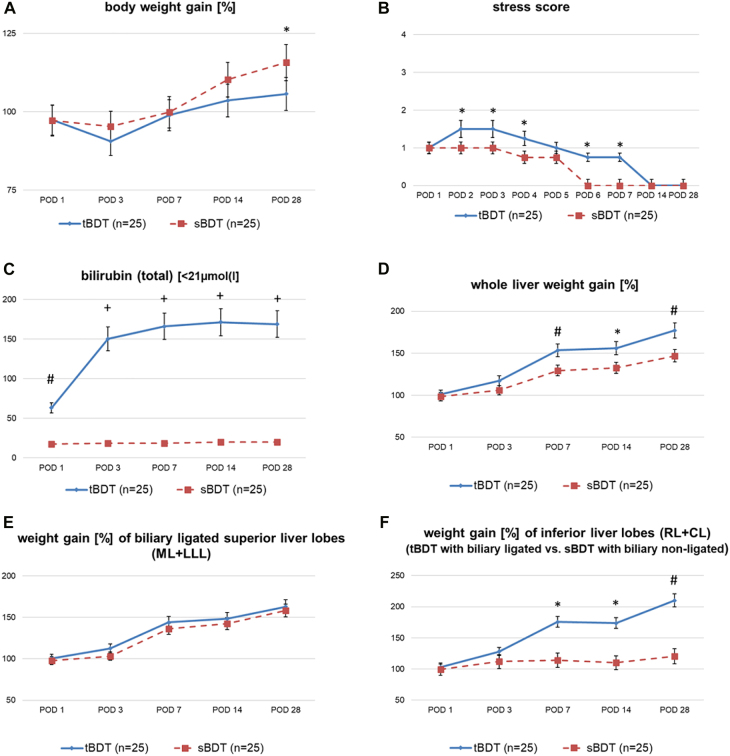
A–F: Results of A: body weight gain, B: stress score; C: bilirubin levels, D: whole liver weight gain following sBDT and tBDT in rats; comparison of differences in weight gain of E: biliary ligated superior liver lobes (ML+LLL) and F: the inferior liver lobes (RL+CL, biliary non-ligated in sBDT) after tBDT and sBDT. (tBDT vs. sBDT same POD: *p<0.05; ^#^p<0.03; ^+^p<0.01).

All animals tolerated the procedure well (survival = 100%). The operation time (“cut-stitching-time”) was significantly longer in sBDT compared to tBDT (min: 42.0 ± 2.5 vs. 28.0 ± 1.7, p=0.04), due to the more complex technique of sBDT (e.g., microsurgery in murine hilar region with placing multiple ligatures) (see Supplementary Tables S2 and S3). We detected no biliary leakage in either group. We determined some ascites in the tBDT animals at POD 28. The rats showed the expected initial weight loss of less than 10% until POD 3 (sBDT: 4.62%; tBDT 9.34%) and recovered their original body weight by POD 8, followed by a constant weight gain throughout the observation period. Animals subjected to tBDT reached about 106% of the original body weight. In contrast, animals subjected to sBDT only reached 116%. Similarly, the daily assessment of the postoperative condition revealed only minor reduction. Again, tBDT caused slightly more stress than sBDT (see Figure 2A–D; and Supplementary Tables S2 and S3).

### Laboratory blood tests results

sBDT caused transient minor liver injury as indicated by a slight elevation of liver enzymes on POD1, but did not cause any alteration of liver or kidney function (e.g., bilirubin total, INR, albumin, creatinine). As expected, sBDT did not lead to elevated bilirubin levels.

In contrast, tBDT caused persisting liver damage with a maximum on POD 1 and slightly decreasing thereafter. As intended, tBDT induced elevation of bilirubin levels, reaching the plateau after POD3 (see Supplementary Tables S2 and S3).

### Liver weight gain

#### After sBDT

We found a substantial increase in whole liver weight reaching about 150% of the standard liver weight due to the volume gain of the biliary ligated (ML+LLL) liver lobes within 4 weeks (see Figure 2D). The biliary ligated liver lobes almost doubled their weight within the 28day observation period (sBDT: ML+LLL at POD 1 with 6.9 g vs. POD 28 with 11.1 g, see Figure 2E). In contrast, the biliary non-ligated (inferior) liver lobes did not show a relevant weight gain (sBDT: CL+RL at POD 1 with 2.9 g vs. POD 28 with 3.6 g) (see Figure 2F).

#### After tBDT

We found a steady increase in whole liver weight gain, reaching almost 2-fold of the starting liver weight within 4 weeks (tBDT: ML+LLL at POD 1 with 7.0 g vs. POD 28 with 11.4 g; CL+RL at POD 1 with 3.0 vs. POD 28 with 6.3 g) (see Figure 2A–F; and Supplementary Tables S2 and S3).

### Histology (HE) and immunohistochemistry (BrdU, EvG)

**Figure 3:**
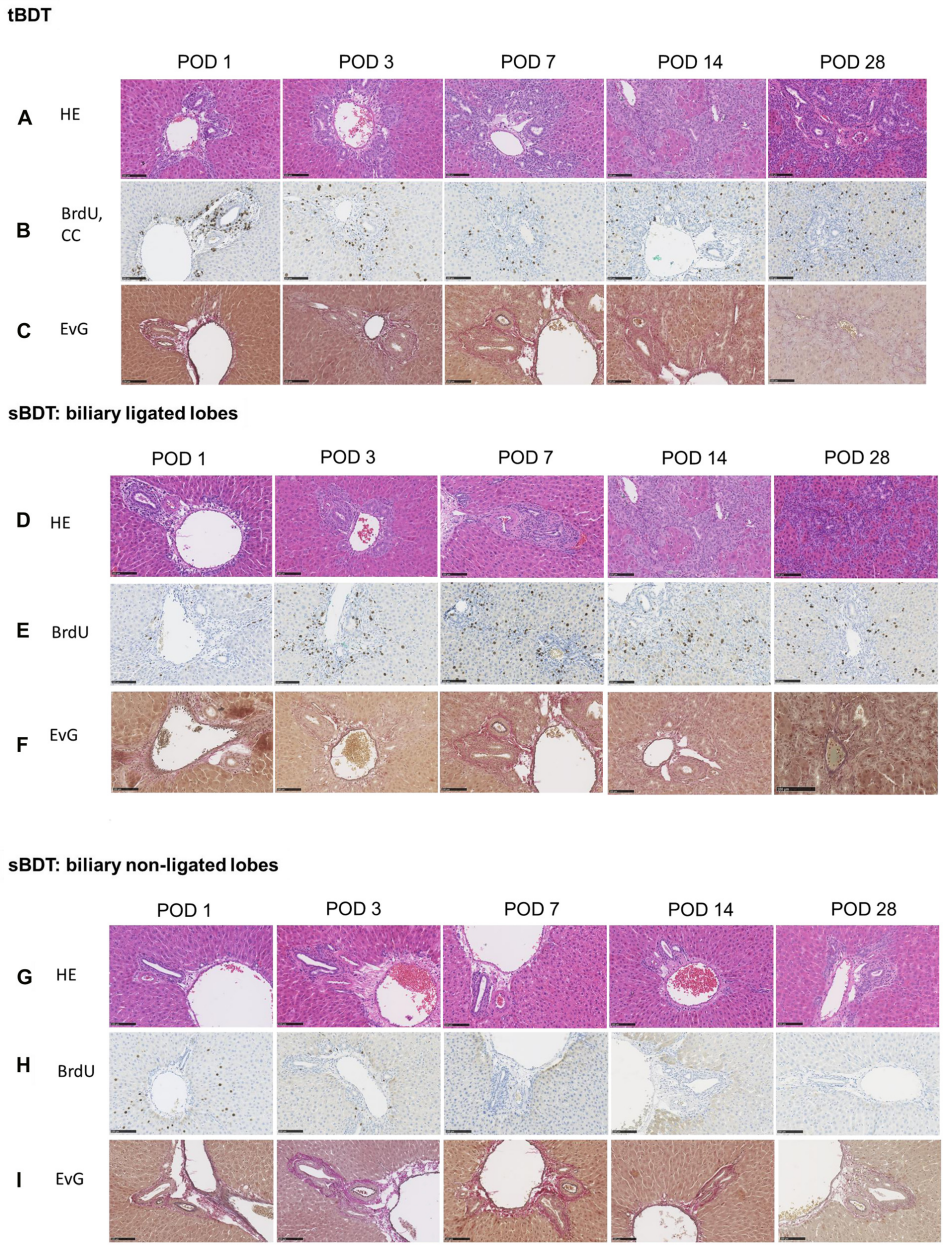
A–I: Histological images following tBDT and sBDT (biliary ligated and non-ligated liver lobes) in rat (in an extra file). The black scale bar represents always 100 µm.

The ductular reaction following biliary occlusion was similar in the biliary ligated liver lobes in both groups (HE, BrdU, EvG). After both procedures, the relative area occupied by biliary proliferates increased and led to a relative reduction in the size of the hepatocellular compartment, a finding corresponding to the relative weight increase in the respective ligated lobes (see Figure 3 and Supplementary Tables S4–6).

### We observed in the biliary non-ligated liver lobes after sBDT a transient increase in biliary proliferates with mild periportal liver fibrosis within the first week

In addition, we determined an accumulation of extracellular collagen peaking at POD 7 followed by a slight decline. This stable increase in fibrous tissue resulted in a persistent mild periportal fibrosis of score 1 (see Figures 3 [Fig j_iss-2021-0044_fig_004] [Fig j_iss-2021-0044_fig_005] [Fig j_iss-2021-0044_fig_006]–7; and Supplementary Tables S4–6). As expected, the extent of the morphological alterations (biliary proliferates in HE, BrdU, EvG) in the biliary non-ligated lobes of the sBDT animals were significantly lower compared to tBDT and the biliary ligated lobes of sBDT animals, respectively.

**Figure 4: j_iss-2021-0044_fig_004:**
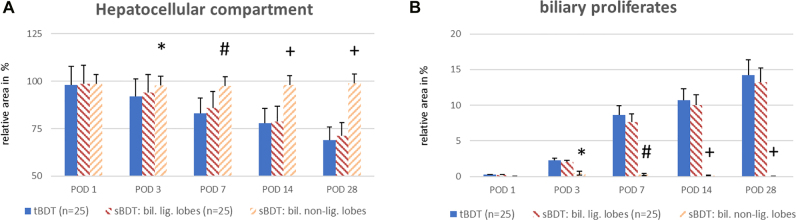
A and B: Hepatic morphometry after sBDT and tBDT in rats. (A) We found a similar relative loss of hepatocellular mass due to the progress of biliary proliferates (B) in the biliary ligated liver lobes in all animals. (sBDT biliary non-ligated liver lobes vs. tBDT and sBDT biliary ligated liver lobes same POD: *p<0.05; ^#^p<0.03; ^+^p<0.01).

**Figure 5: j_iss-2021-0044_fig_005:**
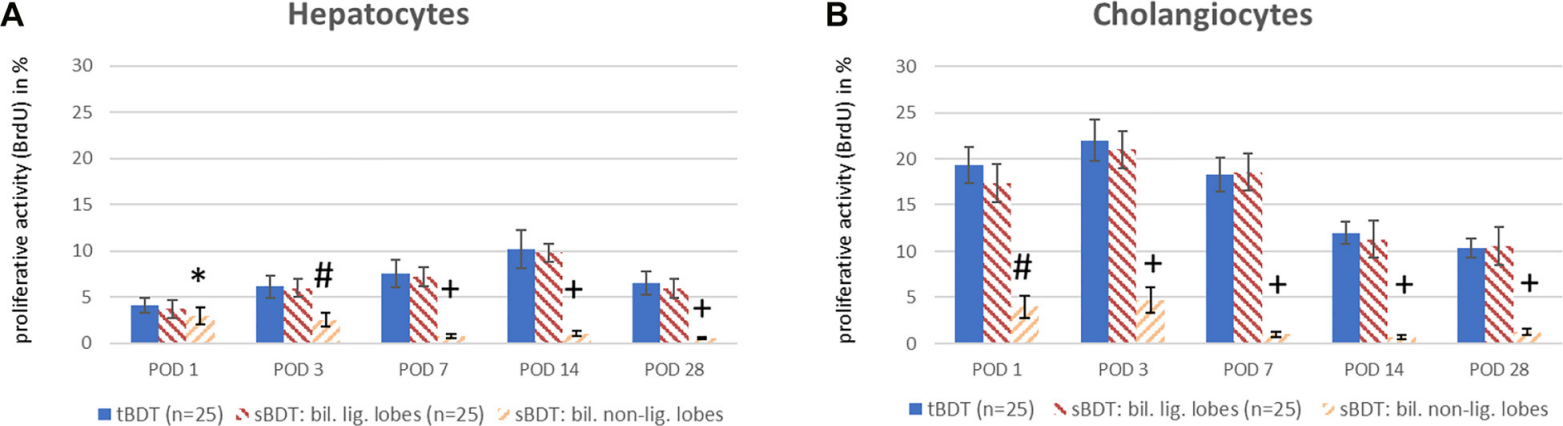
Proliferative activity of hepatocytes and cholangiocytes in rats after sBDT and tBDT. (A) Hepatocellular proliferation (HC) started within 24 h after biliary occlusion, reaching a peak of 10% within 2 weeks and declined to 5% thereafter. (B) Irrespectively of the surgical model, a biliary occlusion caused massive cholangiocellular proliferation (CC) reaching almost 20% within one day after ligation and remained stable throughout the first week, followed by a decline to 10% thereafter. A slight co-reaction of the non-ligated lobes after sBDT was observed reaching a maximum of 5% for CC and a maximum of 3% for HC on day 3. (sBDT biliary non-ligated liver lobes vs. tBDT and sBDT biliary ligated liver lobes same POD: *p<0.05; ^#^p<0.03; ^+^p<0.01).

**Figure 6: j_iss-2021-0044_fig_006:**
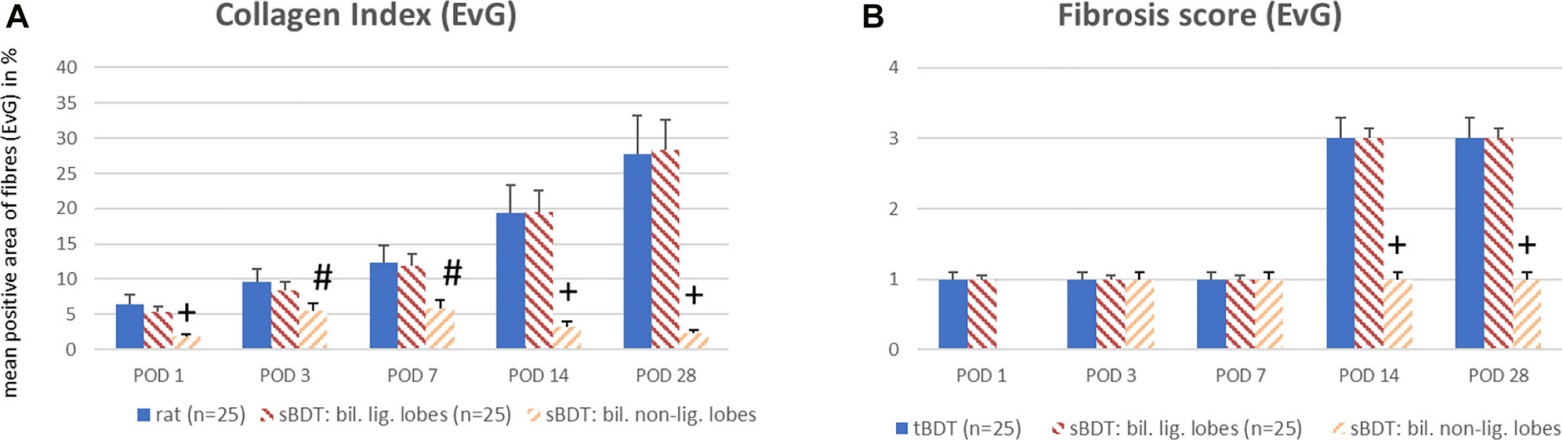
Collagen index and fibrosis score reflecting the zonal distribution of fibrous tissue and severity of fibrosis (Fibrosis score) in rats after sBDT or tBDT (EvG staining). (sBDT biliary non-ligated liver lobes vs. tBDT and sBDT biliary ligated liver lobes same POD: *p<0.05; ^#^p<0.03; ^+^p<0.01).

**Figure 7:**
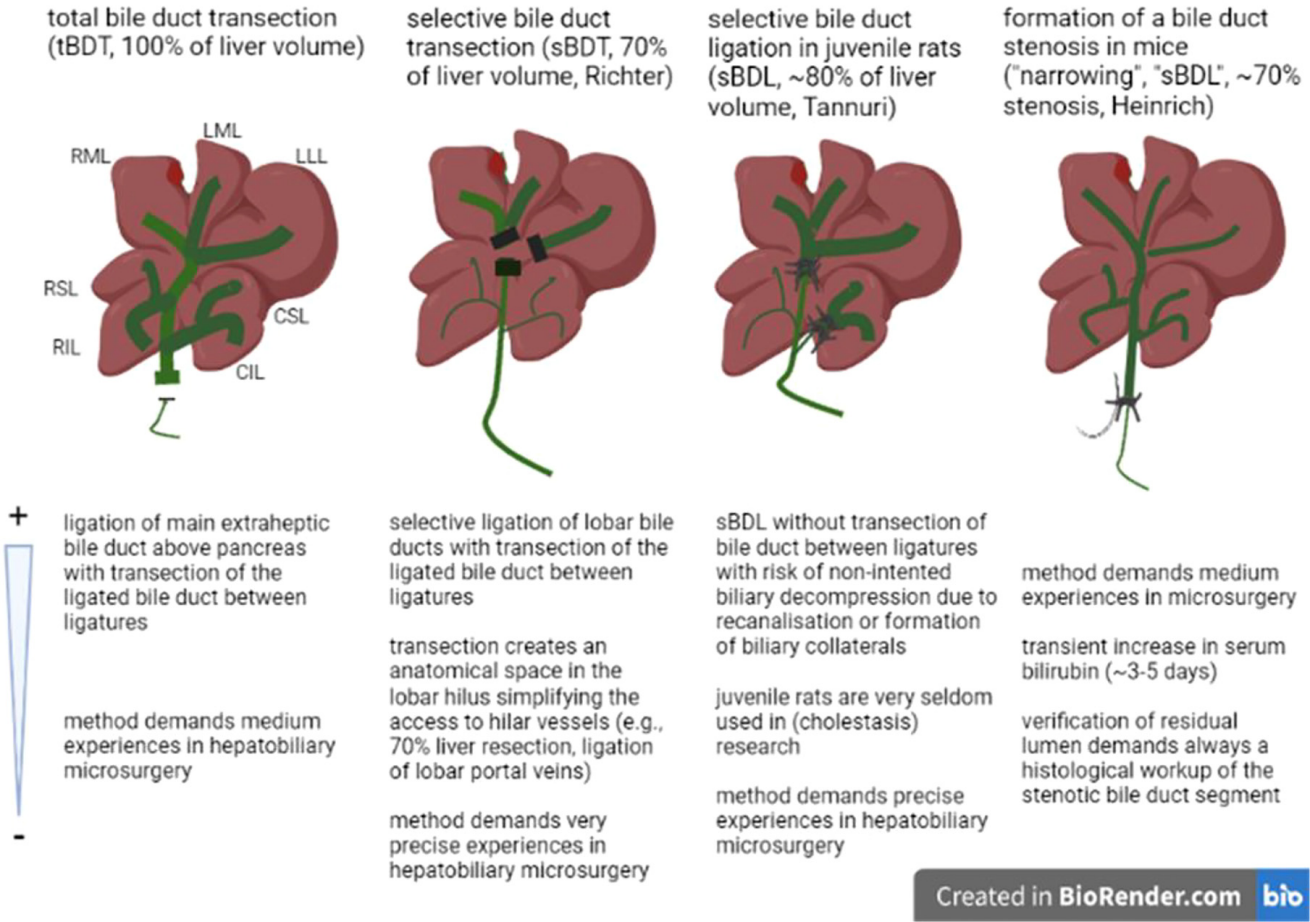
Comparison of different models of total (tBDT) or selective cholestasis (sBDT, sBDL) or subtotal biliary stenosis (“narrowing”) in cholestasis research.

## Discussion

The translation of a clinical problem into an experimental model is still one of the most challenging and interesting aspects in experimental research.

Especially in cholestasis research, only few experimental models for selective biliary occlusion in rats and one model of formation of a subtotal stenosis of the main bile duct in mice were described (see Figure 7) [8, 14, 15].

sBDT is a rather challenging model compared to the other models (tBDT, sBDL and biliary stenosis). Biliary stenosis and tBDT only require manipulation of the common bile duct, but not any delicate steps in the liver hilum. However, few authors reported about substantial mortality of their animals subjected to tBDT and selective biliary occlusion when using sBDL or the tube/needle technique [8–10, 14–16].

For sBDT, the preparation in the lobar hilus for ligation and transection of the dedicated lobar bile duct demands the atraumatic separation from the branches of the portal vein and hepatic artery. Such advanced microsurgical manoeuvres harbour the risk for biliary leakage, bleeding or stenosis of vasculature probably causing hepatic ischemia with an increased risk of mortality of the animals. In addition, these advanced microsurgical manoeuvres can prolong the operation time, probably resulting in an increased rate of anaesthesia related side effects [17, 18]. Therefore, sound experience in microsurgery is needed to ensure stable results. In our study, no animals were lost due to lethal complications. Although sBDT caused a significantly longer operation time compared to tBDT, we only noticed minor impairment of the animal condition and a minor weight loss in the sBDT animals during the first three days. In the stenosis model, no lobar bile duct branches were ligated or transected, but the extrahepatic common bile duct was subjected to formation of a segmental stenosis (“narrowing”) [8, 14]. Mostly, extra fine, small tubes or surgical needles were placed beneath the common bile duct during ligation. The tubes or needles were used to assure a subtotal stenosis (e.g., 70–90% stenosis) of the ligated bile duct segment [8, 14]. The extent of stenosis was histologically proven in one publication [14]. The mortality among the mice was 19–33% [15]. The authors described or discussed no need for special training in hepatobiliary microsurgery for their technique [8, 14]. Another model comprised ligation of lobar bile duct branches in juvenile rats [15]. The biliary branches were occluded with two ligatures, but not transected between the ligatures. The authors described bleeding from the liver surface and anaesthetic side effects in juvenile rats as main causes for the mortality of 17% of their animals [15]. The authors emphasized that operative interventions in the liver hilus and in juvenile animals need precise experiences in hepatobiliary microsurgery. In our model, sBDT induced a stable ductular reaction in the biliary ligated lobes leading to a gain in liver lobe weight and volume, comparable to the changes observed after tBDT. Similar results were only described in the model of sBDL in juvenile rats by Tannuri [15]. The authors of the “stenosis model” described a temporary increase in serum bilirubin and a ductular reaction in histology within the first week, followed by a rather rapid decline of the systemic and histological parameters of cholestasis [14]. The authors recommended their model for further investigation of resolving acute cholestasis.

We detected a transient biliary reaction with a persistent mild periportal fibrosis in the biliary non-ligated liver lobes in all sBDT animals. Since we inducted the obstructive cholestasis far away and “upstream” from the inferior and biliary non-ligated liver lobes, we can exclude accidental impairment of the bile drainage of the non-ligated liver lobes. The literature describes transient or late “cholestatic alterations” of liver tissue after severe ischemia and prolonged onset of reperfusion for several diseases, predominantly in human (e.g., severe polytrauma, sepsis, liver transplantation ± use of marginal organs) [19–22]. However, our model did not include any key aspects of ischemia and reperfusion injury (IRI) and did not affect the blood supply or drainage of the non-ligated liver lobes, besides the short-time anaesthesia for the induction of sBDT or tBDT. We used the established inhalative anaesthesia with isoflurane, since this has become the standard in experimental research [23]. In addition, we found no signs for severe hepatocellular damage, necrosis or ischemia in the ligated or non-ligated liver lobes in either group within the 4 weeks observation period. Therefore, it seems rather unlikely that the manipulation at the superior segments of the bile duct could have interfered with the far away located hilar vessels of the inferior liver lobes. So, it seems not reasonable to search within the complex cascade of IRI for a potential explanation for the transient “cholestatic co-reaction”. Moreover, several anatomic studies addressed the potential for biliary recanalization or formation of biliary collaterals after ligation of the main bile duct with a single or double ligatures (“BDL”). The studies detected different incidences of biliary recanalization, but found no evidence for the existence of (extra-anatomic) interlobar bile ducts that served as biliary bypass leading to biliary decompression of the biliary obstructed liver lobes in rodents [16, 24–29]. Again, we found no signs for a (transient or persistent) biliary decompression in the biliary ligated liver lobes after tBDT and sBDT as indirect indicator for non-intended biliary decompression leading to a transient ductular reaction in the biliary non-ligated lobes in sBDT. Since the systemic bilirubin (total) level was in normal range in all sBDT animals, the potential influence of bilirubin as a paracrine-like mediator seems also not very convincing. More likely seems to be the explanation that the pro-proliferative mediator cascade initiated in the biliary ligated liver lobes due to sBDT might have stimulated the “transient co-reaction of the biliary non-ligated liver lobes”. This explanation is supported by the short-time (POD1-3) increased proliferative activity of hepatocytes and cholangiocytes in the biliary-non ligated liver lobes after sBDT.

As the diverse genetically altered mice lines offer a very broad spectrum for further analysis, the transfer of sBDT into mice is an absolute desirable but also still challenging aim in cholestasis research. From the anatomical view point, the technique of sBDT should be transferable to the mouse anatomy. Since hepatobiliary microsurgery in mice demands much more precise technical experiences and anatomical knowledge than in rats (e.g., organ size, resistance to stress, anaesthesia, and operation time), this model should predominantly (only) be used by extremely well experienced microsurgeons (including their lab-teams).

However, the literature gives no information about similar findings in human cholestatic livers. Since the focus of the study laid on basic cholestatic research, we cannot present evidences for this hypothesis at the moment. We do believe that the transient cholestatic alterations in the biliary non-ligated liver lobes were stimulated by such paracrine interlobar mechanism.

## Conclusions

Our model of sBDT represents a safe and valid method for inducing a lobar limited cholestasis, when performed by experienced microsurgeons with precise knowledge of the murine liver anatomy. The model opens the opportunity for simultaneous investigation of liver regeneration in biliary obstructed and biliary non-obstructed liver lobes including examinations for potential interlobar mediators leading to different hepatobiliary remodelling.

## Supplementary Material

Supplementary Material

Supplementary Material

Supplementary Material
