# Supplementary material for: Selective biliary occlusion in rodents: description of a new technique
Source: Innov Surg Sci. 2022 Jun 23;7(1):13–22. doi: 10.1515/iss-2021-0044 (PMC9352182; doi:10.1515/iss-2021-0044)
Supplement: Supplementary file 2 — Supplementary Material [file j_iss-2021-0044_suppl_002.pdf]

Supplement

Material and Methods:

Surgical techniques

All surgical interventions were performed at day-time under inhalation of 2-3% isoflurane mixed with pure oxygen at a flow rate of 0.5L/min (isoflurane vaporizer, Sigma, UK) in a dedicated S1 operation room. All instruments were thoroughly cleaned and tip-sterilized between the operations. At the end of the day the instruments were cleaned and sterilized in a commercial autoclave (Systec, Germany). All procedures were done using an operating microscope (Zeiss, magnification 10-25x, Germany) to ensure preservation of the branches of the hepatic artery and portal vein.

Preoperative preparation of the animals:

All animals were weighed and anaesthetized with 3% isoflurane and 0.5 L/min oxygen in an induction chamber. The abdomen was shaved, and animals were placed in a supine position on a small animal operation table and fixed with tape. The anaesthesia was maintained as described above. The abdominal skin was disinfected with iodine solution. A sterile operation field was created by placing sterile gauzes around the disinfected skin. A transverse incision was made in the upper third of the abdomen. Closure of the abdominal wound was always done by two-layer running suture (Prolene 6-0, Ethicon).

Postoperative care and analgesic treatment of the animals:

Analgesic treatment was started immediately after the wound closure in all animals. Buprenorphine (0.05mg/kg BW, Temgesic®) was injected subcutaneously; twice per day for the first three postoperative days. During this time the animals were checked for their clinical condition also twice per day; afterwards the animals were routinely checked once per day. For postoperative monitoring, the rats were weighed daily. Clinical scoring was performed according to Hawkins [1].

Experimental groups

sBDT (selective ligation and transection of the bile duct inducing cholestasis in 70% of liver volume: median lobe and left lateral lobe):

For *sBDT* (~70% cholestasis) each two silk ligatures were placed (silk 6-0) around the bile duct draining the median lobe (ML), then around the bile duct draining the left lateral lobe (LLL), and finally around the distal segment of the superior part of the main bile duct. The ligated bile duct was transected distal to the ligatures of ML and LLL. We

used this complex ligature and transection technique to prevent biliary leakage and non-intended recanalization of the bile duct.

The median lobe (ML) and the left lateral lobe (LLL) account for 70% of liver volume, whereas the non-ligated, remaining liver lobes consisted of the right lobes (RL) and caudate lobes (CL) account for 30% of liver volume [5,6].

tBDT (ligation and transection of the main bile duct inducing cholestasis in 100% liver volume) at POD 0:

For *tBDT* (~100% cholestasis) three ligatures were placed around the main extrahepatic bile duct ca. 1cm above the pancreas. The ligated main extrahepatic bile duct was transected between the middle and most distal ligature.

Sacrifice at PODs 1, 3, 7, 14, 28:

One hour prior to sacrifice the animals were injected with 50mg BrdU/kg body weight. After relaparotomy, blood samples were taken from the infrahepatic vena cava. The animals were sacrificed by exsanguation under anaesthesia. The liver was explanted, weighed and the volume of the remnant liver and single liver lobes were determined. Samples of all liver lobes were collected for histological and immunohistochemical analysis according to a standardized protocol.

Haematoxylin-eosin staining (HE)

The samples were fixed in 4.5% buffered formalin for 48h. Sections of 4µm thickness were cut after paraffin embedding. Slides were stained with Haematoxylin-Eosin (HE) for histo-pathological examination. After staining, all slides were digitalized using a slide scanner (Nanozoomer 2.0 HT scanner and the software NDP.scan 2.3; Hamamatsu City, Japan).

Number, size and relative area of necrotic areas or abscesses were evaluated with the measuring tool of NDP-Viewer ("NanoZoomer Digital Pathology"; Hamamatsu, Japan). Results for the number of necrotic areas were given as numerical value, for size in mm^2, and relative size in %. The relative area represents the area of necrosis in relation to area of the total section [%].

Number and relative area of periportal fields as well as the ductular reaction (bile ducts in portal area and extraportal convolutes of bile ducts) were determined and given as described for necrotic areas.

Bromodeoxyuridine (BrdU Staining)

The staining procedure was based on a modified protocol of Sigma Inc. After deparaffinization and rehydration, tissue sections were treated with prewarmed 0.1% trypsin solution at 37°C for 20 minutes, followed by denaturation with 2 N HCl at 37°C for 30 minutes, and blocking with avidin solution for 10 minutes, biotin solution for 10 minutes, and 5% goat serum BSA-TBS at 37°C for 15 minutes. In the next step sections were incubated with 1:50 monoclonal anti-BrdU antibody (DAKO Inc.) at 37°C for 1 hour, followed by 1:300 biotinylated Fab-specific goat anti-mouse linked antibody (Sigma Inc.) for 30 minutes and AP-conjugated streptavidin (DAKO Inc.) for 30 minutes, prior to the application of Neofuchsin solution for 20 minutes. The sections were washed, counterstained with Hematoxylin, and coverslipped with Immu-Mount (Shandon Inc.).

Elastica-van-Gieson (EVG)

Formalin-fixed paraffin-embedded liver biopsy tissues were sliced to a thickness of 4 µm and underwent Elastica van Gieson (EVG) staining using the following procedure: Deparaffinized and hydrated sections were dipped in 70% ethanol containing 1% hydrogen chloride, incubated in resorcin–fuchsin solution for 60 minutes, and washed in 100% ethanol and in water, followed by counterstaining with van Gieson's solution (saturated picric acid containing 0.09% acid fuchsin) for 5 minutes, and coverslipped with Immu-Mount (Shandon Inc.).

Reference for Supplement:

1. Hawkins P. Recognizing and assessing pain, suffering and distress in laboratory animals Laboratory Animals 2002 36, 378–395.
