# Supplementary material for: Selective biliary occlusion in rodents: description of a new technique
Source: Innov Surg Sci. 2022 Jun 23;7(1):13–22. doi: 10.1515/iss-2021-0044 (PMC9352182; doi:10.1515/iss-2021-0044)
Supplement: Supplementary file 3 — Supplementary Material [file j_iss-2021-0044_suppl_003.pdf]

Table 2:
Laboratory chemistry. survival and weight data **after tBDT** in rats (n=5 per time point).
The body weight gain and liver-weight-body-weight ratio were calculated with the starting body weight.
(tBDT vs. sBDT same POD: * p<0.05; # p<0.03; + p<0.01)

	**POD 1**			**POD 3**			**POD 7**			**POD 14**			**POD 28**		
	mean	±	STDV	mean	±	STDV	mean	±	STDV	mean	±	STDV	mean	±	STDV
**Laboratory Chemistry**															
ASAT [<0.83 µmol/l.s]	16.83	±	6.32	10.29	±	1.92	7.12	±	1.18	6.91	±	1.59	8.38	±	1.95
ALAT [<0.74 µmol/l.s]	12.00	±	4.67	5.67	±	1.63	2.33	±	0.53	1.60	±	0.38	3.66	±	2.49
Bilirubin (total) [<21 µmol/l]	63.17	±	7.54 #	150.25	±	18.86+	166.13	±	27.84+	171.40	±	13.38+	168.86	±	20.51+
Albumin [33-53 g/l]	6.17	±	0.69 +	6.25	±	0.43 +	6.50	±	0.87 +	5.40	±	0.49 +	5.60	±	0.49 +
Glucose [3.9-5.8 mmol/l]	7.37	±	0.87	6.13	±	0.58	7.65	±	1.05	6.86	±	1.05	6.18	±	1.21
Creatinine [35-100 µmol/l]	26.50	±	2.93	28.50	±	1.50	29.25	±	1.85	29.80	±	3.06	27.40	±	1.36
INR [71-120%]	117.90	±	2.81	118.27	±	3.28	117.82	±	4.01	119.01	±	2.77	118.38	±	1.35
**Survival. weight data. operation time**															
survival at time point [number; %]	5/5		100%	5/5		100%	5/5		100%	5/5		100%	5/5		100%
body weight gain [%]	97.39	±	2.01	90.66	±	2.86	98.91	±	3.21	103.61	±	1.98	105.72	±	2.37 *
liver weight [g]	10.11	±	0.32	11.71	±	0.45	15.35	±	0.35 #	15.60	±	0.34 *	17.71	±	0.34 #
superior liver lobes (ML+LLL. ~70% liver)	7.03	±	0.31	7.88	±	0.42	10.08	±	0.43	10.38	±	0.36	11.41	±	0.35
inferior liver lobes (CL+RL. ~30% liver)	3.08	±	0.34	3.83	±	0.47	5.27	±	0.32 *	5.21	±	0.21 *	6.31	±	0.37 #
liver-body weight ratio [%]	3.70	±	0.32	4.17	±	0.27	5.59	±	0.32*	6.39	±	1.11 *	6.81	±	0.78 *
spleen weight [g]	0.52	±	0.03	0.58	±	0.06	0.87	±	0.18	0.73	±	0.23	1.06	±	0.21 *
operation time ("cut-stitching-interval) [min]	28	±	1.7 *												

Table 3:
Laboratory chemistry. survival and weight data **after sBDT** in rats (n=5 per time point).
The body weight gain and liver-body-weight ratio (lbwr) were calculated with the starting body weight.

	**POD 1**			**POD 3**			**POD 7**			**POD 14**			**POD 28**		
	mean	±	STDV	mean	±	STDV	mean	±	STDV	mean	±	STDV	mean	±	STDV
**Laboratory Chemistry**															
ASAT [<0.83 µmol/l.s]	20.32	±	17.39	12.42	±	4.57	6.15	±	3.11	7.39	±	5.86	10.31	±	3.18
ALAT [<0.74 µmol/l.s]	15.59	±	5.16	8.74	±	3.26	5.35	±	2.45	1.02	±	0.75	0.92	±	0.52
Bilirubin (total) [<21 µmol/l]	17.24	±	7.18	18.32	±	6.29	18.22	±	5.93	19.89	±	4.46	19.91	±	4.51
Albumin [33-53 g/l]	33.21	±	5.49	35.01	±	1.99	36.19	±	3.91	37.71	±	7.31	35.13	±	5.81
Glucose [3.9-5.8 mmol/l]	8.36	±	1.12	7.99	±	0.95	9.37	±	0.89	7.10	±	1.87	9.01	±	3.12
Creatinine [35-100 µmol/l]	67.51	±	6.31	77.18	±	13.29	82.81	±	7.24	78.97	±	11.24	75.91	±	7.01
INR [71-120%]	110.81	±	2.82	109.54	±	3.21	112.00	±	4.71	116.19	±	4.29	117.19	±	6.01
**Survival. weight data. operation time**															
survival at time point [number; %]	5/5		100%	5/5		100%	5/5		100%	5/5		100%	5/5		100%
body weight gain [%]	97.20	±	3.12	95.38	±	3.21	99.86	±	5.97	110.32	±	8.91	115.76	±	4.51
liver weight [g]	9.83	±	0.31	10.58	±	0.32	12.95	±	0.37	13.27	±	0.34	14.69	±	0.39
superior liver lobes (ML+LLL. ~70% liver. biliary ligated)	6.85	±	0.31	7.23	±	0.21	9.53	±	0.39	9.97	±	0.35	11.08	±	0.41
inferior liver lobes (CL+RL. ~30% liver. biliary non-ligated)	2.98	±	0.27	3.35	±	0.32	3.42	±	0.36	3.30	±	0.29	3.61	±	0.32
liver-body-weight-ratio [%]	3.52	±	0.17	3.60	±	0.35	3.64	±	0.32	3.56	±	0.39	3.75	±	0.31
spleen weight [g]	0.50	±	0.04	0.63	±	0.03	0.68	±	0.06	0.56	±	0.07	0.60	±	0.08
operation time ("cut-stitching-interval) [min]	42	±	2.5												

Table 4
Morphometry and Immunohistochemistry (BrdU, EvG) of liver tissue **after tBDT** in rats (n=5 per time point).

	POD 1			POD 3			POD 7			POD 14			POD 28		
	mean	±	STDV	mean	±	STDV	mean	±	STDV	mean	±	STDV	mean	±	STDV
**Portal fields (PF)**															
relative area of portal fields [%]	1.76	±	1.21	5.69	±	1.37	8.29	±	1.03	11.34	±	2.79	16.87	±	2.42
number of portal fields	13.29	±	2.85	15.03	±	1.51	14.00	±	3.78	14.16	±	2.58	13.57	±	3.62
number of bd per PF	7.35	±	2.31	22.38	±	7.35	29.13	±	2.81	35.05	±	9.50	80.31	±	6.78
diameter of bd per PF [µm]	5.30	±	1.32	16.54	±	3.24	20.09	±	3.68	29.80	±	5.14	33.18	±	4.67
**Extraportal ductular reaction**															
relative area [%]	0.25	±	0.72	2.26	±	0.98	8.61	±	1.21	10.68	±	1.67	14.21	±	2.53
number of biliary convolutes	5.38	±	1.32	12.75	±	2.31	14.85	±	3.11	15.74	±	7.07	21.09	±	8.31
number of BD per convolute	3.4	±	1.31	13.98	±	1.26	51.09	±	2.01	65.3	±	6.56	88.91	±	5.89
diameter of bd per convolute [µm]	2.56	±	1.57	12.58	±	1.83	16.13	±	3.21	18.31	±	5.78	21.45	±	3.94
**Hepatocytes**															
relative area [%]	97.98	±	1.25	92.04	±	1.93	82.94	±	1.73	77.83	±	1.64	68.92	±	3.64
**Necrosis**															
number	0.3	±	0.72	0.41	±	0.53	1.69	±	0.78	1.18	±	0.25	0	±	0
relative area [%]	0.01	±	0.51	0.01	±	0.31	0.16	±	0.42	0.15	±	0.36	0	±	0
**Proliferation-Index (BrdU)**															
hepatocytes	4.13	±	2.82	6.12	±	2.39	7.54	±	2.81	10.2	±	3.8	6.52	±	3.17
cholangiocytes	19.34	±	6.87	22.01	±	7.37	18.31	±	2.93	12.00	±	3.21	10.39	±	3.28
**EvG**															
Collagen Index	6.45	±	1.76	9.59	±	1.89	12.32	±	2.01	19.37	±	3.97	27.66	±	9.51
Fibrosis score	1			1			1			3			3		

Table 5
Morphometry and Immunohistochemistry (BrdU, EvG) of the **biliary ligated liver lobes after sBDT** (n=5 per time point).

	POD 1			POD 3			POD 7			POD 14			POD 28		
	mean	±	STDV	mean	±	STDV	mean	±	STDV	mean	±	STDV	mean	±	STDV
**Portal fields (PF)**															
relative area of portal fields [%]	1.12	±	0.68	3.92	±	1.27	6.34	±	2.77	10.99	±	4.65	15.62	±	6.24
number of portal fields	12.63	±	2.94	13.87	±	2.99	15.31	±	2.96	14.33	±	3.19	14.97	±	4.81
number of bd per PF	6.27	±	2.31	21.99	±	6.29	28.39	±	4.71	34.04	±	4.62	78.91	±	5.31
diameter of bd per PF [µm]	4.26	±	1.85	15.52	±	5.97	19.71	±	3.21	28.09	±	4.99	31.62	±	8.34
**Extraportal ductular reaction**															
relative area [%]	0.22	±	0.91	2.01	±	0.64	7.61	±	1.32	9.98	±	1.99	13.21	±	3.25
number of biliary convolutes	4.26	±	0.21	10.96	±	2.81	13.27	±	3.71	15.23	±	4.62	22.51	±	3.21
number of bd per convolute	2.75	±	1.01	12.06	±	1.37	48.82	±	8.09	62.91	±	8.32	84.97	±	4.45
diameter of bd per convolute [µm]	2.39	±	1.86	11.97	±	4.21	15.82	±	2.91	17.92	±	4.98	20.01	±	5.07
**Hepatocytes**															
relative area [%]	98.65	±	1.72	94.06	±	5.24	85.90	±	3.76	78.89	±	4.35	71.17	±	5.01
**Necrosis**															
number	0.26	±	0.89	0.31	±	0.87	1.67	±	0.65	1.08	±	0.52	0	±	0
relative area [%]	0.01	±	0.52	0.01	±	0.29	0.15	±	0.4	0.14	±	0.42	0	±	0
**Proliferation-Index (BrdU)**															
hepatocytes	3.71	±	2.81	5.99	±	3.01	7.21	±	0.65	9.81	±	4.21	5.94	±	3.52
cholangiocytes	17.33	±	6.31	20.98	±	8.43	18.56	±	2.87	11.31	±	3.21	10.53	±	3.31
**EvG**															
Collagen Index	5.31	±	2.43	8.41	±	1.43	11.84	±	3.01	19.58	±	4.01	28.41	±	9.61
Fibrosis score (median)	1			1			1			3			3		

Table 6
Morphometry and Immunohistochemistry (BrdU, EvG) of the **biliary non-ligated liver lobes after sBDT** (n=5 per time point).
(sBDT biliary non-ligated liver lobes vs. tBDT and sBDT biliary ligated liver lobes same POD: * p<0.05; # p<0.03; + p<0.01)

	POD 1			POD 3			POD 7			POD 14			POD 28		
	mean	±	STDV	mean	±	STDV	mean	±	STDV	mean	±	STDV	mean	±	STDV
**Portal fields (PF)**															
relative area of portal fields [%]	1.02	±	0.57	1.78	±	1.27 #	2.35	±	2.01 #	1.89	±	3.25 +	1.09	±	2.96 +
number of portal fields	12.36	±	2.49	13.03	±	2.84	12.39	±	2.87	11.35	±	4.31	12.64	±	5.58
number of bd per PF	2.14	±	0.98 *	4.23	±	1.31 #	4.67	±	2.57 +	3.86	±	3.99 +	5.17	±	3.95 +
diameter of bd per PF [µm]	4.72	±	1.52	4.33	±	2.87 #	3.97	±	3.99 +	4.02	±	2.74 +	3.96	±	2.91 +
**Extraportal ductular reaction**															
relative area [%]	0.01	±	0.14 *	0.50	±	0.83 #	0.3	±	0.73 +	0.12	±	0.54 +	0.03	±	0.39 +
number of biliary convolutes	2.11	±	0.21	3.29	±	1.82 #	3.23	±	3.25 +	2.09	±	3.49 +	1.51	±	2.09 +
number of bd per convolute	2.45	±	0.78	2.76	±	1.56 #	3.02	±	1.03 +	2.98	±	1.73 +	2.54	±	1.83 +
diameter of bd per convolute [µm]	2.61	±	1.82	4.28	±	2.99 #	3.89	±	2.6 +	3.97	±	2.59 +	3.01	±	2.89 +
**Hepatocytes**															
relative area [%]	98.97	±	1.72	97.72	±	5.24 *	97.35	±	3.76 #	97.99	±	4.35 +	98.88	±	5.01 +
**Necrosis**															
number	0	±	0 *	0	±	0 *	0	±	0 #	0	±	0 #	0	±	0
relative area [%]	0	±	0 *	0	±	0 *	0	±	0 #	0	±	0 #	0	±	0
**Proliferation-Index (BrdU)**															
hepatocytes	2.98	±	1.06 *	2.52	±	0.97 #	0.78	±	0.48 +	1.02	±	1.01 +	0.53	±	0.74 +
cholangiocytes	3.98	±	0.72 #	4.71	±	0.87 +	1.01	±	0.97 +	0.71	±	1.01 +	1.23	±	0.99 +
**EvG**															
Collagen Index	1.78	±	1.43 +	5.49	±	1.32 #	5.81	±	2.46 #	3.29	±	2.95 +	2.34	±	1.95 +
Fibrosis score (median)	0			1			1			1		+	1		+
