# Supplementary material for: Selective biliary occlusion in rodents: description of a new technique
Source: Innov Surg Sci. 2022 Jun 23;7(1):13–22. doi: 10.1515/iss-2021-0044 (PMC9352182; doi:10.1515/iss-2021-0044)
Supplement: Supplementary file 1 — Supplementary Material [file j_iss-2021-0044_suppl_001.pdf]

## Reviewer Assessment

# B Richter et al.: Selective biliary occlusion in rodents: Description of a new technique

## Reviewers' Comments to Original Submission

**Reviewer 1: anonymous**

Date received: 27-Jan-2022
Reviewer recommendation: **Return to author for minor modifications**
Reviewer overall scoring: **High**

Assessment Form scores: 5 = High/Yes; 3 = Medium/Adequate; 1 = Low

| | | | | | |
|---|---|---|---|---|---|
| Is the subject area appropriate for the journal | 5 | | | | |
| Does the title clearly reflect the paper's content? | 5 | | | | |
| Does the abstract clearly reflect the paper's content | 5 | | | | |
| Do the keywords clearly reflect the paper's content? | | 4 | | | |
| Does the introduction present the problem clearly? | 5 | | | | |
| Are the results/ conclusions justified? | | 4 | | | |
| How comprehensive and up-to-date is the subject matter presented? | | 4 | | | |
| How adequate is the data presentation? | | | 3 | | |
| Are units and terminology used correctly? | | 4 | | | |
| Is the number of cases adequate? | 5 | | | | |
| Are the experimental methods/ clinical studies adequate? | 5 | | | | |
| Is the length appropriate in relation to the content? | 5 | | | | |
| Does the reader get new insights from the article? | 5 | | | | |
| Please rate the practical significance. | 5 | | | | |
| Please rate the accuracy of methods. | 5 | | | | |
| Please rate the statistical evaluation and quality control. | | | | | 1 |
| Please rate the appropriateness of the figures and tables. | | 4 | | | |
| Please rate the appropriateness of the references. | | 4 | | | |
| Please evaluate the writing style and use of language. | 5 | | | | |
| Please judge the overall scientific quality of the manuscript. | | 4 | | | |
| Are the methods used worthy of reproduction in greater deal? | Yes | | | | |
| Would you be willing to review a revision of this manuscript? | Yes | | | | |

**Comments to author:** The present study describes the establishment of a novel animal model of selective biliary occlusion in rats. For this purpose, the common bile duct or only the left bile duct were ligated and transected in 50 rats. Throughout an observation period of 28 days, the outcome of the two procedures was assessed by measuring a broad spectrum of histomorphological, regenerative and systemic parameters.

This is a well performed study, which extensively characterizes the herein introduced novel model of selective cholestasis and, thus, provides a solid basis for the further use of this innovative experimental approach. In addition, the manuscript is well written and the content is clear. Congratulations to the authors!

I have only a few minor comments, which need further clarification:
1. Statistical analysis: It is not described, which statistical tests were used, although the authors describe several times "a significant difference". Moreover, statistical differences are neither indicated in the graphs nor the tables. Please carefully address this issue and include the necessary information throughout the entire manuscript and data sets, where appropriate.
2. Results, page 6, lines 24-25: Presented data should always be given with identical precision. E.g. "42+/-2.5" should read "42.0+/-2.5"
3. Results, page 6, lines 36-37: "Animals subjected to tBDT reached about 110% of the origi-nal body weight. In contrast, animals subjected to sBDT only reached 105%". This state-ment does not correspond to the data shown in Fig. 1A.
4. Discussion: It would be interesting to discuss, whether the herein described surgical ap-proach would be also transferable to mice, which offer a much broader spectrum of trans-genic lines for mechanistic analyses – or is this too challenging?
5. Discussion, page 12, lines 27 and 31: Repetition of the word "Therefore" should be avoid-ed.
6. Figures 2: Please include clearly labeled scale bars. The information "20x" for the magnifi-cation makes no sense.
7. Several text passages in the results and discussion as well as in the figure legends are in bold, which makes no sense at all. Please correct the layout.

**Reviewer 2:** anonymous

Date received: 27-May-2020
Reviewer recommendation: **Accept in present form**
Reviewer overall scoring: **High**

Assessment Form scores: 5 = High/Yes; 3 = Medium/Adequate; 1 = Low

| | | | | | |
|---|---|---|---|---|---|
| Is the subject area appropriate for the journal | | 4 | | | |
| Does the title clearly reflect the paper's content? | 5 | | | | |
| Does the abstract clearly reflect the paper's content | 5 | | | | |
| Do the keywords clearly reflect the paper's content? | 5 | | | | |
| Does the introduction present the problem clearly? | 5 | | | | |
| Are the results/ conclusions justified? | 5 | | | | |
| How comprehensive and up-to-date is the subject matter presented? | | 4 | | | |
| How adequate is the data presentation? | 5 | | | | |
| Are units and terminology used correctly? | 5 | | | | |
| Is the number of cases adequate? | 5 | | | | |
| Are the experimental methods/ clinical studies adequate? | 5 | | | | |
| Is the length appropriate in relation to the content? | 5 | | | | |
| Does the reader get new insights from the article? | 5 | | | | |
| Please rate the practical significance. | | 4 | | | |
| Please rate the accuracy of methods. | 5 | | | | |
| Please rate the statistical evaluation and quality control. | | 4 | | | |
| Please rate the appropriateness of the figures and tables. | 5 | | | | |
| Please rate the appropriateness of the references. | 5 | | | | |
| Please evaluate the writing style and use of language. | | | | | |

| | | | | | |
|---|---|---|---|---|---|
| Please judge the overall scientific quality of the manuscript. | 5 | | | | |
| Are the methods used worthy of reproduction in greater deal? | Yes | | | | |
| Would you be willing to review a revision of this manuscript? | Yes | | | | |

**Comments to author:** This is a very extensive and elaborate study. The question is highly relevant because hypertrophy concepts are increasingly used in liver surgery, especially for the treatment of pCCC patients.

The paper is well written and the methodology well explained. The number of experimental animals in the groups seems sufficient. Of particular interest is the finding that bile duct proliferation occurs initially on the non-bile duct occluded side.

## Authors' Response to Reviewer Comments

Date received: 08-Mar-2022

### Response to reviewer 1

Comments to the Author
The present study describes the establishment of a novel animal model of selective biliary occlusion in rats. For this purpose, the common bile duct or only the left bile duct were ligated and transected in 50 rats. Throughout an observation period of 28 days, the outcome of the two procedures was assessed by measuring a broad spectrum of histomorphological, regenerative and systemic parameters.

This is a well performed study, which extensively characterizes the herein introduced novel model of selective cholestasis and, thus, provides a solid basis for the further use of this innovative experimental approach. In addition, the manuscript is well written and the content is clear. Congratulations to the authors!
I have only a few minor comments, which need further clarification:

1. Statistical analysis: It is not described, which statistical tests were used, although the authors describe several times "a significant difference". Moreover, statistical differences are neither indicated in the graphs nor the tables. Please carefully address this issue and include the necessary information throughout the entire manuscript and data sets, where appropriate.
*At first we thank you very much for your very kind statements.*
*We thank you very much for your important comment. We want to apologize for the incomplete presentation of the data. We have corrected the text and inserted signs for significant differences in the Tables and Figures.*

2. Results, page 6, lines 24-25: Presented data should always be given with identical precision. E.g. "42+/-2.5" should read "42.0+/-2.5"
*We have corrected the text according to your recommendations.*

3. Results, page 6, lines 36-37: "Animals subjected to tBDT reached about 110% of the origi-nal body weight. In contrast, animals subjected to sBDT only reached 105%". This state-ment does not correspond to the data shown in Fig. 1A.
*We thank you very much for your remark. We want to apologize for the incorrect wording we used in the description of the results. We have corrected the text.*

4. Discussion: It would be interesting to discuss, whether the herein described surgical ap-proach would be also transferable to mice, which offer a much broader spectrum of trans-genic lines for mechanistic analyses – or is this too challenging?
*We thank you very much for your interesting request. Comparing the liver anatomy of rats and mice, the technique of sBDT should be transferable to the mouse anatomy. Since hepatobiliary microsurgery in mice demands much more precise technical experiences and anatomical knowledge than in rats (e.g., organ size, stress resistance, anaesthesia, operation time), this model should predominantly (only) be used by extremely well experienced microsurgeons (including their lab-teams). We do agree with*

*your statement, that the diverse mice lines offer a very broad spectrum for further analysis. Consequently, the transfer of sBDT into mice is an absolute desirable but also still challenging aim in cholestasis research.*

*We inserted a small paragraph for this special topic in the discussion.*

5. Discussion, page 12, lines 27 and 31: Repetition of the word "Therefore" should be avoid-ed.
*We changed the sentence according to your recommendations.*

6. Figures 2: Please include clearly labeled scale bars. The information "20x" for the magnifi-cation makes no sense.
*We thank you for your important request. We have included scale bars in the histopictures (always representing 100µm).*

7. Several text passages in the results and discussion as well as in the figure legends are in bold, which makes no sense at all. Please correct the layout.
*We apologize for the inconveniences we made by marking special passages and subheadings in bold. We have changed these passages into a normal text layout. Since we compare two different operative groups within the manuscript, we would like to recommend to keep the marking of either group (e.g., sBDT or tBDT, biliary ligated or biliary non-ligated liver lobes) within the Tables for better discrimination between the groups or treated liver areas.*

**Response to reviewer 2**

Comments to the Author

This is a very extensive and elaborate study. The question is highly relevant because hypertrophy concepts are increasingly used in liver surgery, especially for the treatment of pCCC patients.

The paper is well written and the methodology well explained. The number of experimental animals in the groups seems sufficient. Of particular interest is the finding that bile duct proliferation occurs initially on the non-bile duct occluded side.

*We thank you very much for your very kind comments.*

*We agree with you that the initially co-reaction of the biliary non-ligated liver lobes to the sBDT of the residual liver lobes is one of the interesting finding of this study. At the moment we can not give a concrete explanation for this finding. We do believe, that the sBDT of 70% of liver volume might have induced a temporary proliferation stimuli to the residual 30% (biliary non-ligated) liver lobes resulting in the transient cholestatic alterations.*

## Comments by the Editor-in-Chief to Revised Submission – Final Decision

All reviewer comments were addressed by the authors. Reviewer raised only minor points for improvement on the original version. Therefore, I accept the revised manuscript for publication in our journal.
